# P-2260. Incidence and clinical characteristics of acute viral infections in the first year after hematopoietic stem cells transplantation. Argentine single center cohort

**DOI:** 10.1093/ofid/ofae631.2413

**Published:** 2025-01-29

**Authors:** Emilio F Huaier Arriazu, Diego H Giunta, Ana L Basquiera, Noelia Mañez, Julieta M De Boeck, Emilse D Diaz Lobo, Martin A Rolan, Mariángeles Visús, Marisa del Lujan Sanchez

**Affiliations:** Hospital Italiano de Buenos Aires, Ciudad Autónoma de Buenos Aires, Ciudad Autonoma de Buenos Aires, Argentina; Centre for Pharmacoepidemiology (CPE) - KI, and Hospital Italiano de Buenos Aires, Stockholm, Stockholms Lan, Sweden; Hospital Privado Universitario de Córdoba, Cordoba, Cordoba, Argentina; Hospital Italiano de Buenos Aires, Sede Central., Buenos Aires, Ciudad Autonoma de Buenos Aires, Argentina; Hospital Italiano de Buenos Aires, Ciudad Autónoma de Buenos Aires, Ciudad Autonoma de Buenos Aires, Argentina; Hospital Italiano de Buenos Aires, Ciudad Autónoma de Buenos Aires, Ciudad Autonoma de Buenos Aires, Argentina; Hospital Italiano de Buenos Aires, Ciudad Autónoma de Buenos Aires, Ciudad Autonoma de Buenos Aires, Argentina; Hospital Italiano de Buenos Aires, Sede Central., Buenos Aires, Ciudad Autonoma de Buenos Aires, Argentina; Hospital Italiano de Buenos Aires, Ciudad Autónoma de Buenos Aires, Ciudad Autonoma de Buenos Aires, Argentina

## Abstract

**Background:**

The incidence and mortality of respiratory virus infections (RVI) in patients receiving a Hematopoietic Stem Cell Transplant (HCT) is variable, and changes depending on place, year and season. They can present as upper respiratory tract infection (URTI) or lower respiratory tract infection (LRTI), with the latter having a worse prognosis. There is no data from Argentina in the adult population. Objectives: estimate the incidence of RVI in the year after HCT and associate risk factors for clinical evolution, progression from URTI infection to LRTI, requirement for Intensive Care Unit (ICU) and mortality.

Flow chart of HCT recipients and respiratory events, with a diagnosis of respiratory viral infection within one year after transplant.

**Figure:**
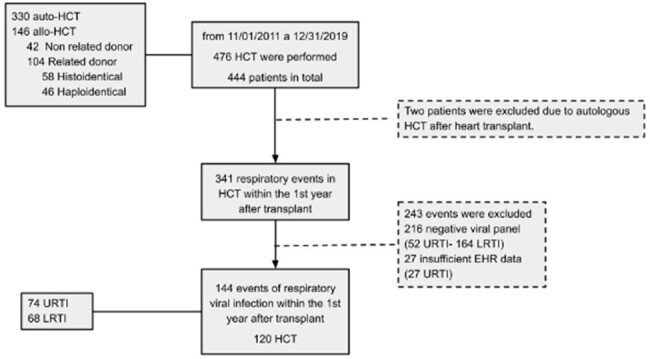

**Methods:**

retrospective cohort study of the population of patients receiving HCT from 11/2011 to 12/2019. The patients were followed for one year, and for one month after each RVI.

Kaplan-Meier curve of the incidence of IRVA according to type of HCT.

**Figure:**
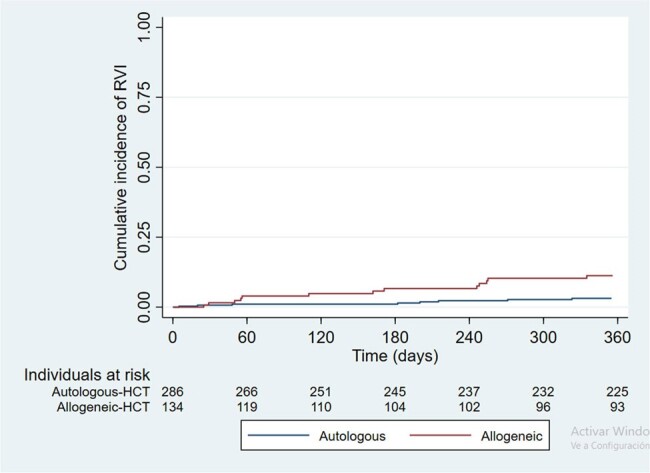

**Results:**

Out of 476 HCT with 30.7% allogeneic, 99 confirmed RVI were detected. The Cumulative Incidence at 100 days post-transplant was 1.99 (95% CI 1,0 - 3,95) per 100 patients, while at one year, it was 28.81 per 100 patients (95% CI 24.32 - 33). ,93). The HR of RVI in allogeneic HCT was 2.45 (95% CI 1.72-3.51; p < 0.001) compared to autologous. Also was lower in lymphoma 0.5 (95% CI 0.29 - 0.85) and Multiple Myeloma 0.36 (95% CI 0.22 - 0.58) compared to leukemia (p < 0.001) as the underlying disease. Although there were differences in the clinical status and characteristics evaluated at the diagnosis of RVI, no relevant associations were found at day 7 of follow-up. 53.5% of RVI events presented as URTI, and 16.9% progressed to LRTI. An association was found between male sex, OR of 0.20 (95% CI 0.06-0.73; p< 0.001) and the occurrence of the event beyond 28 days after transplant, 0.27 (95% CI 0.08 -0.95; p=0.045), while RVI in hospitalized patients had an OR of 5.71 (95% CI 1.61-20.24; p=0.006) for progression. Only 16% required ICU, there was an association between age over 65 years, event before 28 days post HCT, myeloablative conditioning, use of corticosteroids at the time of the event, RVI during hospitalization and hypoxemia and requiring ICU. Mortality was 2.1% (95% CI 0.67-6.32) within 30 days of RVI.

**Conclusion:**

The Cumulative Incidence was similar to other reports, however the mortality was lower.

**Disclosures:**

All Authors: No reported disclosures

